# The Role of Socioeconomic Status in Longitudinal Trends of Cholera in Matlab, Bangladesh, 1993–2007

**DOI:** 10.1371/journal.pntd.0001997

**Published:** 2013-01-10

**Authors:** Elisabeth Dowling Root, Joshua Rodd, Mohammad Yunus, Michael Emch

**Affiliations:** 1 Department of Geography and Institute of Behavioral Science, University of Colorado at Boulder, Boulder, Colorado, United States of America; 2 ICDDR,B, Centre for Health and Population Research, Dhaka, Bangladesh; 3 Department of Geography and Carolina Population Center, University of North Carolina at Chapel Hill, Chapel Hill, North Carolina, United States of America; University of California San Diego School of Medicine, United States of America

## Abstract

There has been little evidence of a decline in the global burden of cholera in recent years as the number of cholera cases reported to WHO continues to rise. Cholera remains a global threat to public health and a key indicator of lack of socioeconomic development. Overall socioeconomic development is the ultimate solution for control of cholera as evidenced in developed countries. However, most research has focused on cross-county comparisons so that the role of individual- or small area-level socioeconomic status (SES) in cholera dynamics has not been carefully studied. Reported cases of cholera in Matlab, Bangladesh have fluctuated greatly over time and epidemic outbreaks of cholera continue, most recently with the introduction of a new serotype into the region. The wealth of longitudinal data on the population of Matlab provides a unique opportunity to explore the impact of socioeconomic status and other demographic characteristics on the long-term temporal dynamics of cholera in the region. In this population-based study we examine which factors impact the initial number of cholera cases in a *bari* at the beginning of the 0139 epidemic and the factors impacting the number of cases over time. Cholera data were derived from the ICDDR,B health records and linked to socioeconomic and geographic data collected as part of the Matlab Health and Demographic Surveillance System. Longitudinal zero-inflated Poisson (ZIP) multilevel regression models are used to examine the impact of environmental and socio-demographic factors on cholera counts across *baris*. Results indicate that baris with a high socioeconomic status had lower initial rates of cholera at the beginning of the 0139 epidemic (*γ_01_* = −0.147, p = 0.041) and a higher probability of reporting no cholera cases (*α_01_* = 0.156, p = 0.061). Populations in *baris* characterized by low SES are more likely to experience higher cholera morbidity at the beginning of an epidemic than populations in high SES *baris*.

## Author Summary

Cholera is a bacterial disease usually spread through contaminated water that causes severe diarrhea and dehydration. Modern sewage and water treatment have virtually eliminated cholera in industrialized countries but cholera is still present throughout much of SE Asia, Latin America and sub-Saharan Africa. One of the reasons cholera is still problematic is that genetically distinct forms of the bacteria (often called biotypes) have developed and spread rapidly because the population has no natural immunity to the new biotype. In Bangladesh, the 0139 biotype developed in 1993 and caused a large epidemic. Although it is widely accepted that poor conditions place people at risk for cholera, very few studies have examine what role low socioeconomic status plays in cholera risk, especially during a new epidemic of the disease. In this paper, we explore how local-level socioeconomic status, measured using assets, education and sanitation, affect the severity of the cholera outbreak experienced during the O139 epidemic in Matlab, Bangladesh. We believe our study highlights the importance of improving overall socioeconomic status, not just sanitation and water treatment, in controlling the spread of cholera.

## Introduction

Despite global efforts to improve drinking water quality and sanitation in developing countries, cholera continues to pose a serious public health problem. In 2010, 317,534 cases were reported from 48 countries, an increase of 130% from just 10 years earlier [1]. Many of the cases in 2010 were reported in Haiti, marking the first time since 1995 that the majority of worldwide cases were from the Americas [1]. Globally, the true number of cholera cases is much higher and there is serious under-reporting due to poor surveillance systems and fear of trade sanctions and lost tourism [2]. Cholera remains a global threat to public health and a key indicator of lack of socioeconomic development, but ultimate control of cholera depends on overall socioeconomic condition as evidenced in developed countries. Recently, the re-emergence of cholera in some areas of the world has been noted in parallel with the ever-increasing size of vulnerable populations living in poor and unsanitary conditions [2]–[6]. However, most research has focused on cross-sectional cross-country comparisons so that the role of individual- or small area-level socioeconomic status (SES) in cholera dynamics over time has not been carefully studied. This paper describes how SES is related to interannual variability of cholera in rural Bangladesh.

Cholera is endemic in Bangladesh, meaning it is consistently present in the population at relatively low levels. The actual number of cases in the population fluctuates over time, due to a variety of population and environmental factors. Most notably, cholera has a seasonal component, peaking just before and just after the annual monsoons [7], and is related to the suitability of the aquatic environment which naturally supports the *vibrio* bacteria [8]. Fluctuations in natural immunity [9], [10] and population density and proper sanitation [11], [12] also play a role in cholera dynamics. During an epidemic, rates of the disease increase dramatically for a period of time before returning to endemic levels again. When the epidemic is caused by the introduction of a novel serotype, it may take longer for the rates of disease to return to endemic levels because little natural immunity exists in the population. Bangladesh has experienced several epidemics of the disease, at least one of which what caused by the introduction of a new serotype [13]. However, no research to date has examined the impact of local-level SES on cholera dynamics over time or how SES might mediate the impact of a cholera epidemic.

While there is general agreement among researchers that SES plays a role in vulnerability to and transmission of cholera, little research has directly examined the role of individual-or household-level SES in cholera dynamics. Cross-country level analyses indicate that low-income countries have higher rates of cholera than middle- or high-income countries [5] and that cholera rates are related to indicators of literacy, gross national product (GNP) and the Human Development Index ([HDI], a numerical value based on life expectancy, education, and income) [3]. A recent study in Matlab, Bangladesh examined the relationship between cholera occurrence during pre- and post-monsoon epidemics and a local-level SES index and found that SES was an important predictor of cholera occurrence during both periods [6]. While this study included an indicator of local-level SES it did not examine the long-term temporal dynamics of cholera in the region or the direct role of SES during an epidemic.

SES or social class is a complex concept that is often conceptualized as a combination of occupational, educational, financial and locational influences [14]–[16]. Although these dimensions of SES are related, each reflects somewhat different individual and societal forces associated with disease processes. For example, income provides the means by which to purchase assets, adequate housing and food while education provides the skills necessary for acquisition of a job as well as positive social, psychological, and economic resources. Measuring household SES in developing countries poses considerable problems. Standard economic measures of SES use monetary information, such as income or consumption expenditure, which are often unavailable or unreliable and can be time-consuming and challenging to collect [17], [18]. In these settings, the assets a household acquires are a good indicator of their ‘long-run’ economic status [19]–[22]. These asset-based indices often include durable goods (e.g., radio, television, bed, stove) and housing characteristics (e.g., housing material, water and sanitation systems). Additional measures of social status, such as education and employment, are not as problematic to collect, though may not show much variation in certain locations (e.g., areas where everyone is engaged in subsistence agriculture).

Reported cases of cholera in Matlab, Bangladesh have fluctuated greatly over time and epidemic outbreaks of cholera continue, most recently with the introduction of a new serotype (*V. cholerae* 0139) into the region in 1993. The rich longitudinal data collected on the population of Matlab provides a unique opportunity to explore the impact of SES and other demographic characteristics on the long-term temporal dynamics of cholera in the region. We suggest that in the presence of a new serotype, we can investigate the importance of socioeconomic and socio-demographic factors in determining the severity of the initial outbreak of the disease. In this study we use longitudinal multilevel models to examine two study questions: 1) what is the effect of SES on the initial number of cholera cases at the beginning of the 0139 epidemic and, 2) what is the effect of SES on the trajectory of decline in cholera cases over the subsequent 15 years? We hypothesize that *baris* with lower overall SES will experience a greater number of cholera cases at the beginning of the study period, and that the decline in cases in these low SES *baris* over time will occur more slowly than high SES *baris*.

## Methods

### Study area

Matlab is located in south-central Bangladesh approximately 50 km south-east of Dhaka. Most residents of Matlab are engaged in agricultural production, though increasingly young men and women migrate to Dhaka for work in the textile industry. The study area is 184 km^2^, and is divided into 2 nearly equal portions by the Dhonagoda River. Matlab is densely populated with about 1,200 people per square kilometer, and a total population of nearly 225,000 [23]. Rural Bangladeshis live in groups of patrilineally-related households called *baris*. *Baris* are located on raised plots of land surrounded by agricultural fields, and *bari* members interact closely and typically share water sources (wells and ponds) and latrine facilities. An average of six distinct households constitute a *bari* and the different households in a *bari* are typically comprised of related individuals.

### Data

Identification and surveillance of cholera cases in Matlab has been ongoing since 1964 when data collection began in conjunction with several early cholera vaccine trials. Detailed demographic, socioeconomic and disease data are currently collected by the International Center for Diarrheal Disease Research, Bangladesh (icddr,b). The icddr,b was preceded by the Pakistan-SEATO Cholera Research Laboratory, which collected surveillance data prior to the establishment of icddr,b. In this study, we only use data from 1993 onward and icddr,b has been responsible for cholera surveillance and health and demographic surveillance system activities since this time. The icddr,b maintains a hospital at their Matlab research site which is well known as a regional diarrhea treatment center. Patients admitted with diarrhea are tested at the on-site laboratory for cholera, shigellosis and other pathogenic causes of diarrheal disease. From the icddr,b health records, we obtained data on 3,541 laboratory-confirmed cholera cases that occurred between January 1, 1993 (the year 0139 was introduced) and December 31, 2007. All cases of cholera that occurred during the study period were eligible for inclusion in the study. These cholera laboratory data were linked to the Matlab Health and Demographic Surveillance System (MHDSS), a comprehensive demographic surveillance system also maintained by icddr,b which contains a unique ID for the *bari* within which each individual lives. Detailed information on the MHDSS is available elsewhere [23], [24]. The *bari* was used as the unit of analysis in this study because oral-fecal transmitted diseases often affect several households in a *bari* because of close contact and sharing of resources among households within a *bari*. Cholera cases were assigned to the *bari* location from which they occurred, creating a *bari*-year dataset which contained a count of cholera cases in each *bari* for each year between 1993 and 2007. Cholera cases for which no *bari* was recorded – either due to reporting error or because the patient lived outside Matlab – were excluded from the analysis.

From the MHDSS we obtained the total population count and mean age of each *bari* for each year from 1993 to 2007. All *baris* in existence the study area between 1993 and 2007 were initially eligible for inclusion in the study. Socioeconomic data are only collected approximately every 10 years when a comprehensive household-level census is taken on the population of Matlab. Although income and consumption measures are not currently available for the population of Matlab, data on household assets, education and sanitation are. Therefore, contextual variables pertaining to household assets and sanitation were obtained from the 1996 and 2005 censuses. Since SES changes occur slowly in Matlab, we felt that the 1996 census data accurately reflected the economic and sanitation conditions in the *bari* at the beginning of the study period, just 3 years earlier, and that the 2005 data accurately reflected the SES conditions for the 3 years at the end of the study period. To create a time-varying predictor of SES, we interpolated the bari-level SES for 1997 through 2004 using linear interpolation methods. We used locational information on each *bari* contained in the Matlab GIS to calculate the distance from each *bari* to the ICDDR,B hospital and the distance to the river. Using Hawth's Tools in ArcGIS v9.3 we also calculated the total population and cholera case count for both 500 and 1,000 meter radius neighborhoods around each *bari*. These variables captured the impact of population density and cholera case load around each *bari*, regardless of the respective sizes of individual *baris*.

Baris were excluded from this analysis if: 1) they did not exists in 1993 (the beginning of the epidemic), 2) no data were recorded for the 1996 census and were therefore missing SES and sanitation variables, and 3) they had fewer than 4 years of data because multilevel longitudinal models provide more stable estimates with three or more years of data. This resulted in a sample of 3,413 cholera cases nested within 7,161 baris for a total of 105,678 observations (*bari*-years). 6,850 of these *baris* (95.7%) had all 15 years of data.

### Socio-economic status measurement

A socioeconomic variable was developed using principal component analysis (PCA) in SAS v9.2, creating a single household-level measure of SES from multiple census variables. The first principle component was the only one with an eigenvalue greater than 1 and captured approximately 41% of the variability in the index measurement. The SES measure reflects a composite of seven dummy variables of ownership of household assets (lamp, quilt, bed, watch, bike, radio, television), two ordinal variables of household wall material and type of latrine facility and one continuous variable of years of education for the head of household (Table 1). The household head could have been either male or female, but the vast majority of cases were male. Where a male head of household was not specified, we used the education of the female head of household. Roof material and ownership of agricultural land, cows and boats were initially included in the PCA but were excluded because they lacked variation across households or did not load with the other variables when creating the factor. SES scores were first developed for each household in the study sample. The household-level SES scores were then collapsed by *bari*, and the mean score represents *bari*-level SES. Both continuous and categorical SES scores were initially included in the models. To create the categorical variable, the bari-level SES scores were sorted from lowest to highest and divided into equal quartiles; higher quartiles reflect higher SES. Ultimately, we chose to include the continuous SES score because the relationship between SES and the outcome was near linear and information is lost when continuous data is converted to a categorical variable.

**Table 1:** Variables included in the Principle Component Analysis (PCA) to create SES Indices.

Household Assets	Wall Material	Latrine Facility	Household Head Education
1 = yes; 0 = no	5 = Pucca/cement	4 = Septic tank/modern toilet	Continuous variable
Bed	4 = Tin	3 = Water sealed/slab latrine	0 (None) to 16 (University)
Lamp	3 = Tin and other material	2 = Open latrine or open place	
Quilt	2 = Bamboo and/or wood	1 = No latrine	
Watch	1 = Other material		
Bike			
Radio			
Television			

We conducted a sensitivity analysis of our SES variable by creating several composite SES variables using different combinations of household asset, years of education and sanitation variables and including each (in combination with sanitation variables) to examine the impact on model results. The first SES variable was a PCA of household assets only. When we included this variable along with the latrine facility variable in the regression analysis, only SES was statistically significant (see example additional models in Table S2). A similar situation occurred when we entered variables for years of education of the male household head or the female household head along with the asset-based SES variable into the models. Given this, we chose to create two additional SES indices: one included latrine facility and the other included latrine facility and education. As a sensitivity analysis, we tested the effects of all three SES indices on cholera outcomes and found near identical results with the strongest effect from the index including assets, education and sanitation. The results of this sensitivity analysis, in our opinion, confirm findings from the literature (presented in the introduction) which suggests that SES is a construct which includes educational, economic and location forces. Thus, we chose to include the SES index with assets, education and sanitation in the final set of models.

### Statistical modeling

The number of cholera cases in each *bari* over time was modeled using multilevel longitudinal zero-inflated Poisson (ZIP) regression models. We chose to model cholera cases using a Poisson distribution rather than creating *bari*-level rates because cholera is a rare disease event, which leads to small numbers and unstable rates which are not normally distributed. The ZIP model allows for a large number of zero cases without compromising the model. Count data, such as the *bari* cholera counts examined in the present study, are often characterized by overdispersion (e.g., the variance is greater than the mean). With rare disease events, overdisperson is often the result of excess zero counts, causing the data to exhibit a bimodal distribution [25]–[27]. Zero-inflated Poisson regression is a method for simultaneously but independently modeling count data that exhibit a bimodal distribution due to both excess zeros and positive counts. These models assume that the data are a mixture of two separate data-generating processes: the first is equivalent to a binary model for prevalence outcome (e.g. cholera cases = 0 or cholera cases >0) while the second process is equivalent to zero-truncated Poisson or negative binomial process. The outcome variable for this second process is the number of cholera cases for those baris where the number of cholera cases >0. Throughout this paper we will discuss the zero-inflated (ZI) and Poisson parts of the models separately. The parameter estimates in the count model test for correlation between variables and increasing counts of cholera. The zero-inflated parameter estimates, in contrast, represent correlation between the variables and a zero count. Thus, the parameter estimates for the count model and the zero-inflated models are typically of opposite signs. Despite the fact that our data were overdispersed, we chose not to use a negative binomial ZIP model (NB ZIP) because the NB ZIP specification did not improve model fit and the random effects portions of the multilevel model accounted for overdispersion.

Longitudinal multilevel models, often called “growth curve” or “growth trajectory” models, examine the change in an outcome (cholera cases) over time [28]. The level-1 component of the multilevel model, also known as the individual growth model, represents the change in cholera cases that each *bari* experiences over the time period under study. It also includes other time varying predictors, such as population density, average age or the interpolated SES value. The level-2 component examines the effect of time-invariant predictors, such as distance to hospital, on between *bari* differences in the change trajectories. Thus, measurements of cholera at different times are nested within *baris*. Just like other multilevel models, longitudinal multilevel models consist of a fixed and a random part. The fixed effects show the shape of each *bari*'s trajectory of change over time and the *bari*'s initial number of cholera cases at the beginning of the study period and the factors that modify these things. The random components of the model allow the value of each *bari*'s growth parameters to vary around these population averages.

The first portion of the ZIP model assesses level and change in the logged Poisson counts of cholera over time. The Poisson portion of our model was specified as:
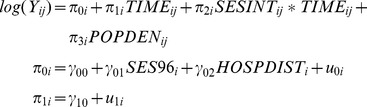
where 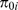 and 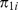 are the *i*th *bari*'s true intercept (initial status) and slope (rate of change), 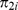 is the difference in the rate of change by level of SES (using interpolated SES), *POPDEN_ij_* is an explanatory variable (population density) for the *i*th *bari* at time *j*, 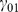 is the population-average effect of SES in 1996 on the intercept (initial status) of the *bari* growth model, *HOSPDIST_i_* is a time invariant explanatory variable of the distance to the icddr,b hospital, and 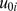 and 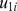 represent *bari*-specific residual terms, which capture variation of each *bari's* intercept and slope around the population average intercept and slope. We assume that covariates are uncorrelated with residuals, and that 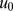 and 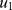 follow a bivariate normal distribution with means of 0 and (co)variances var(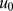), var(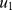), and cov(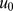,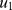).

The second portion of the ZIP model asses the change in zero-inflation and is a growth model based on a logistic regression model. The ZI portion of our model was specified as:
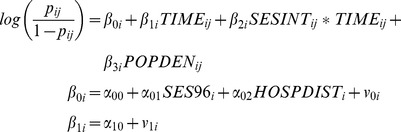
where *p_ij_* is the probability of the *bari* being an inflated zero, 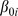 and 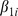 are individual-varying intercepts and slopes, 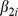 is the difference in the slope by level of SES, and 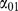 is the population-average effect of SES in 1996 on the intercept, and 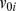 and 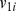 denote the *bari*-level residuals. The aforementioned assumptions about the residuals from multilevel models apply here also (i.e., we assume a fixed scale parameter for the within-subjects model and a bivariate normal distribution for the between-subject residuals).

We conducted both bivariable and multivariable analyses of the data. A variety of different models were fitted but only the final model with the best fit is presented here. Additional covariates we considered include: average age of residents in each *bari*, the cholera rate within a 500-meter and 1000-meter radius buffer around each *bari* (to examine disease environment), average education of the household head and type of latrine facility. We tested the effect of each of these on both the slope and the intercept of the growth model. Two-way interaction terms were also included, but in most instances the equations would not converge or estimates were zero. The age and disease environment variables were not statistically significant in the longitudinal models. All covariates, except the SES index, were centered on the grand mean. The PROC NLMIXED procedure in SAS v9.2 was used for all model estimation.

### Ethics statement

This study was reviewed by the University of North Carolina at Chapel Hill Institutional Review Board and found exempt. All data were anonymized by icddr,b prior to being released to the investigators for analysis.

## Results

**Figure 1:**
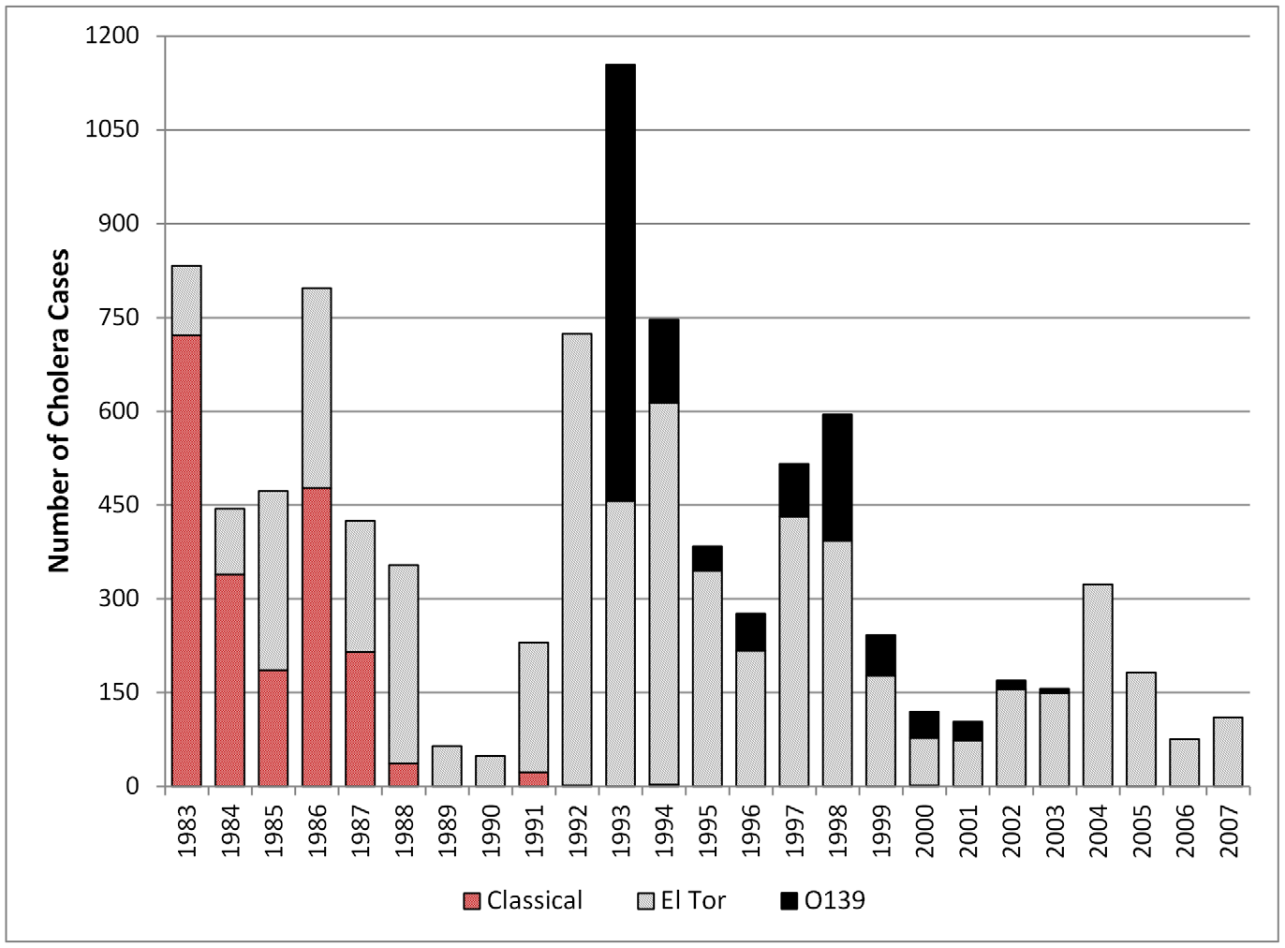
Number of cholera cases, Matlab, Bangladesh, 1983–2007. Stacked bar chart indicating the number of cholera cases by biotype (Classical, El Tor and O139) between 1983 and 2007 in Matlab, Bangladesh. Red bars indicate number of Classical cholera cases, grey bars indicate the number of El Tor cases, and the black bars indicate the number of O139 cases.

**Figure 2:**
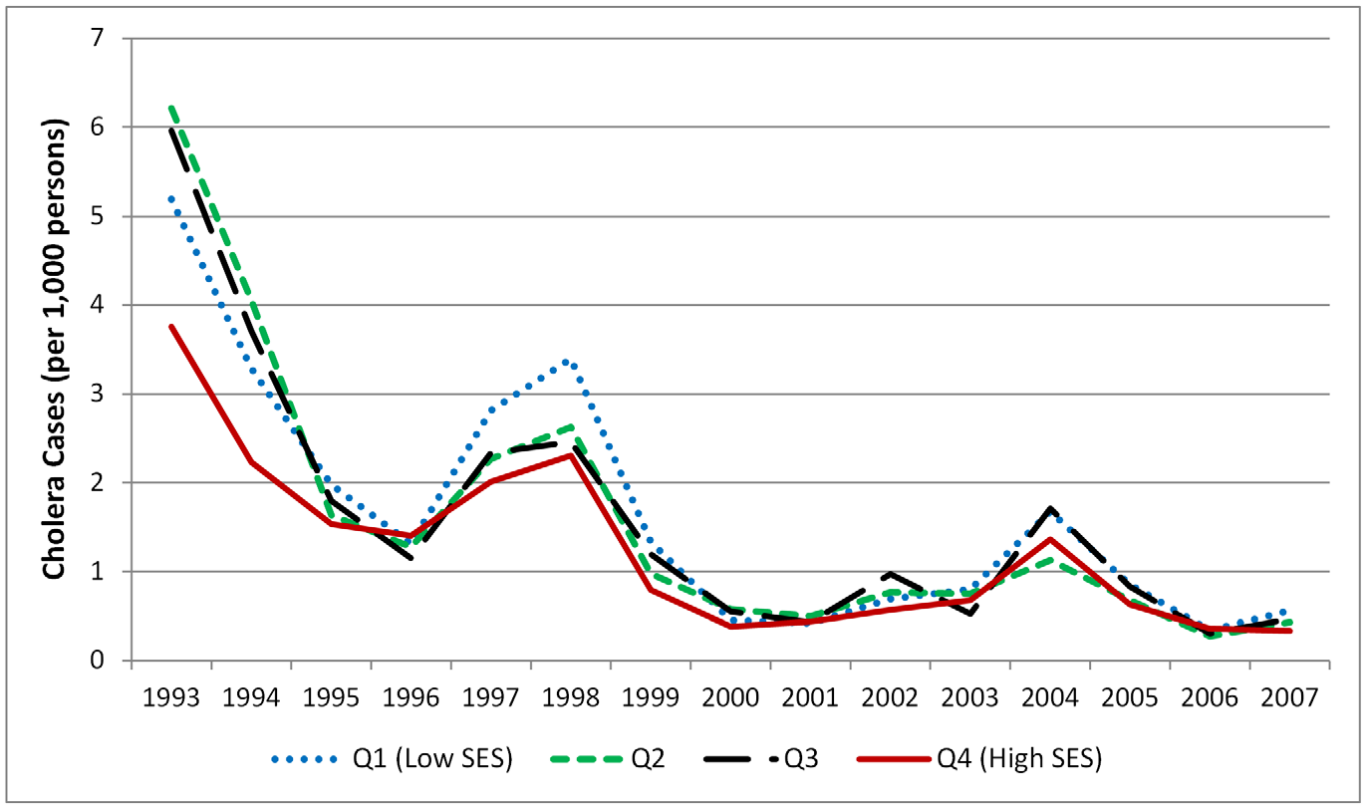
Cholera rate by SES quartile, Matlab, Bangladesh, 1993–2007. Line graph indicating the yearly cholera case rate per 1,000 persons in Matlab, Bangladesh by socioeconomic status index quartile. The blue dotted line indicates SES quartile 1 (the lowest SES) the green dashed line indicates SES quartile 2, the black dashed line indicates SES quartile 3 and the red solid line indicates SES quartile 4 (the highest SES).

Figure 1 shows the total number of cholera cases in Matlab between 1983 and 2007 by cholera biotype (Classical, El Tor and 0139). The figure clearly shows the introduction of 0139 and resulting epidemic peak in 1993 and the decline in cholera cases over the subsequent 15 years. Figure 2 shows the bari-level cholera rates by SES quartile over time. Descriptive statistics for baris with a case of cholera (experienced at any point in time) vs. baris with no reported cases of cholera are shown in Table 2. Of the 7,161 baris included in the analysis, 1,903 (26.5%) reported a cholera case at some point during the study period. Baris with a cholera case had, on average, a larger population and lower SES and were closer to the hospital. Table 3 presents results from the unconditional growth ZIP model and the full random slopes and intercepts models. Since the level-2 random effects for both slope and intercept were not significant in the ZI portion of the model (see Table S1), our final model (Model C) removes these random effects, while keeping the fixed effects, in order to present the most parsimonious model possible.

**Table 2:** Statistical descriptive information of variables used in the analysis.

	*Baris* with a case (n = 1903)			*Baris* with no cases (n = 5258)		
	*N*	*Mean*	*SD*	*N*	*Mean*	*SD*
**Population**						
1993	1903	41.18	33.31	5258	24.4	24.08
2000	1891	41.04	33.21	5163	24.61	24.21
2007	1867	39.67	30.59	5047	24.19	23.49
**Average Age**						
1993	1903	23.76	3.34	5258	24.36	5.03
2000	1891	26.76	3.43	5163	27.39	5.04
2007	1812	28.57	9.77	4585	28.57	12.3
**1996 SES Index**						
Assets only	1891	0.01	0.71	5193	0.15	0.85
Asset, sanitation, education	1891	0.01	0.72	5193	0.16	0.88
**2005 SES Index**						
Assets only	1867	0.00	0.63	5062	0.13	0.73
Asset, sanitation, education	1867	0.00	0.65	5062	0.13	0.76
**Distance to Hospital (meters)**	1903	4876.3	28.4	5258	6599.5	3814.2
**Distance to River (meters)**	1903	1428.0	1120.3	5258	1485.1	1208.9

**Table 3:** ZIP growth curve estimates for cholera trajectories.

	Model A: Unconditional Growth			Model B: Time Invariant SES 96 Modifying Slope and Intercept			Model C: SES 96 Modifying Intercept, Interpolated SES Modifying Slope		
	*Estimate*	*SE*	*p-value*	*Estimate*	*SE*	*p-value*	*Estimate*	*SE*	*p-value*
**Poisson Count Model** (log count)									
**Fixed Effects**									
Intercept (initial status)	−1.938	0.092	<0.0001	−1.187	0.090	<0.0001	−1.195	0.090	<0.0001
SES on intercept				−0.159	0.073	0.0301	−0.147	0.074	0.0417
Time (change)	−0.071	0.017	<0.0001	−0.079	0.013	<0.0001	−0.079	0.013	<0.0001
SES on time				0.015	0.013	0.2343	0.010	0.014	0.4728
Log Population				0.641	0.051	<0.0001	0.659	0.052	<0.0001
Distance to Hospital				−0.058	0.018	0.0010	−0.056	0.017	0.0015
**Variance Components**									
Random Intercept	1.191	0.142	<0.0001	0.544	0.071	<0.0001	0.539	0.070	<0.0001
Random Slope	0.012	0.002	<0.0001	0.008	0.002	<0.0001	0.008	0.002	<0.0001
Covariance	−0.065	0.014	<0.0001	−0.037	0.009	<0.0001	−0.036	0.009	<0.0001
**Zero-Inflated Model** (log odds)									
**Fixed Effects**									
Intercept (initial status)	0.659	0.071	<0.0001	1.490	0.081	<0.0001	1.488	0.082	<0.0001
SES on intercept				0.164	0.082	0.0452	0.156	0.083	0.0612
Time (change)	0.126	0.011	<0.0001	0.121	0.010	<0.0001	0.122	0.010	<0.0001
SES on time				0.004	0.013	0.7640	0.007	0.015	0.6437
Log Population				−0.563	0.052	<0.0001	−0.551	0.054	<0.0001
Distance to Hospital				0.157	0.017	<0.0001	0.159	0.017	<0.0001
−Log Likelihood		33831			31252			30979	
AIC		33845			31282			31009	
BIC		33893			31385			31112	

Results from the unconditional growth model (Model A) indicate that cholera decreased over time. The expected Poisson counts from the model decreased over the 15-year period by about 6.6% per year and the odds of having no cases increased by about 13.4% each year. This equates to an overall rate of change in the ZIP cholera count of approximately −0.1 cases per *bari* per year, or 1.5 cholera cases over the 15 years of the study. There was also significant variation in the random intercept and slope for the Poisson cholera counts, but only significant variation in the random intercept for the ZI portion of the model.

The full final model (Model C) controls for socioeconomic and locational factors that may affect the initial number of cholera cases and the decrease in cases over time. Population has a significant control effect in both the count and zero-inflated part of the model. The positive coefficient in the Poisson portion of the model indicates that a larger *bari* has a higher mean number of cholera cases while the negative coefficient in the ZI portion of the model indicates that the larger a *bari*'s population, the lower the probability of it being cholera-free. In addition, cholera counts decline with increasing distance to the ICDDR,B regional hospital. This is most likely an indication of accessibility problems; people living further from the hospital are less likely to travel to receive treatment for cholera, preferring to administer Oral Rehydration Therapy (ORT) in the home setting. The ZI portion of the model further suggests that as distance to the regional hospital increases, the number of *baris* reporting no cholera cases increases. We also provide an intermediate model (Model B) which shows the effect of SES in 1996 (time invariant) on both the slope and intercept. The AIC, BIC and Log Likelihood scores indicate that this is not the best fit model, and that the time varying measure of SES (interpolated between 1996 and 2005) best measures the modifying effect of SES on the trajectory of cholera over time.

**Figure 3:**
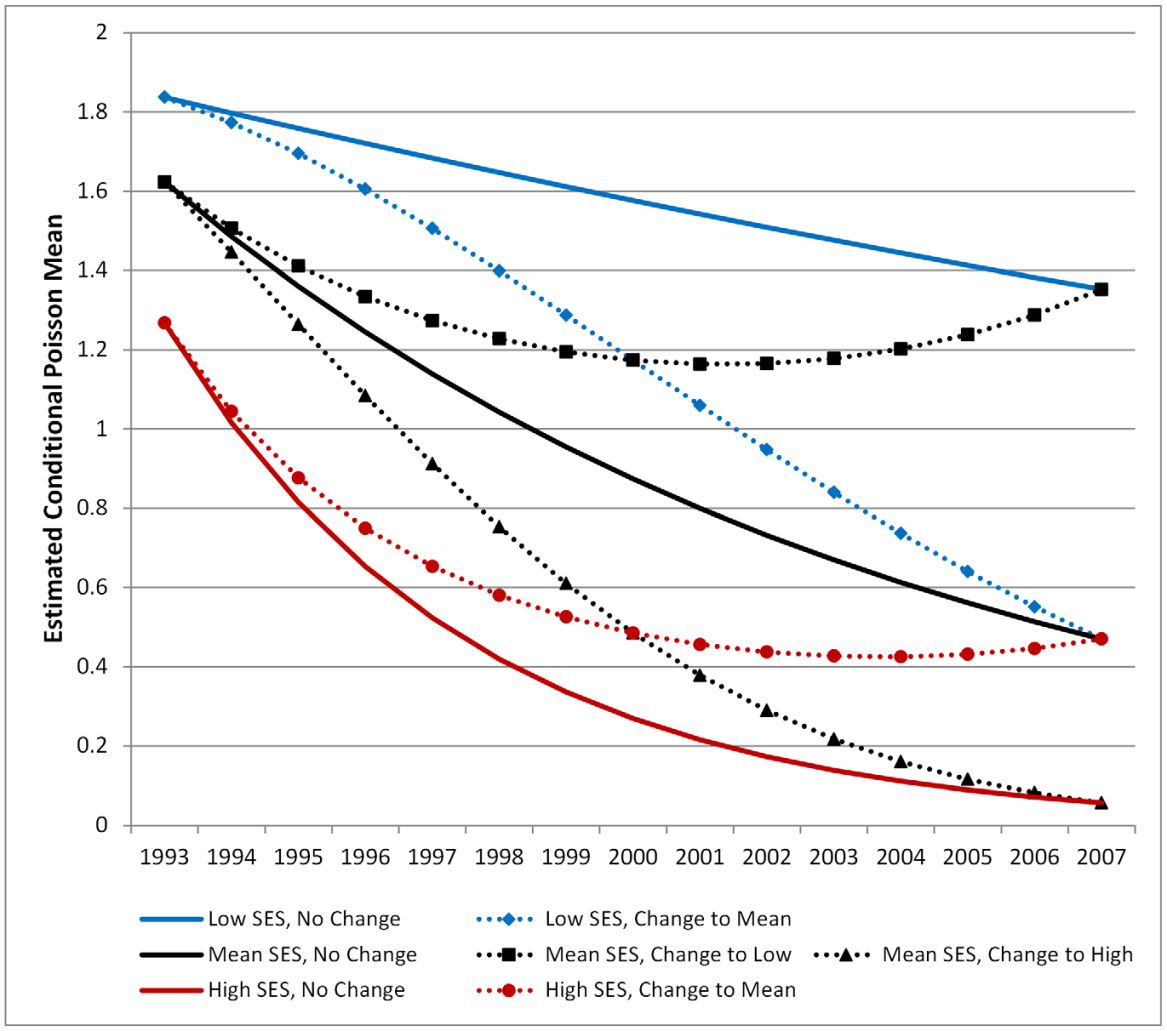
Mean cholera trajectories from Poisson model for different initial SES groups. Line graph indicating the mean trajectory of cholera cases from the conditional Poisson model only (Model C) for different levels of SES. These trajectories were estimated holding population constant at 40 people per *bari* and distance from the icddr,b hospital at 5 km. The blue lines model the trajectory of cholera for *baris* with low initial SES (1 SD below the mean), the black lines model cholera for *baris* with mean initial SES, and the red lines model cholera for *baris* with high initial SES (1 SD above the mean). Solid lines indicate trajectories for *baris* where the level of SES stays constant over the study period. Dotted lines indicate trajectory for *baris* where the level of SES either increases or decreases over time. The figure legend indicates how SES changes over time.

**Figure 4:**
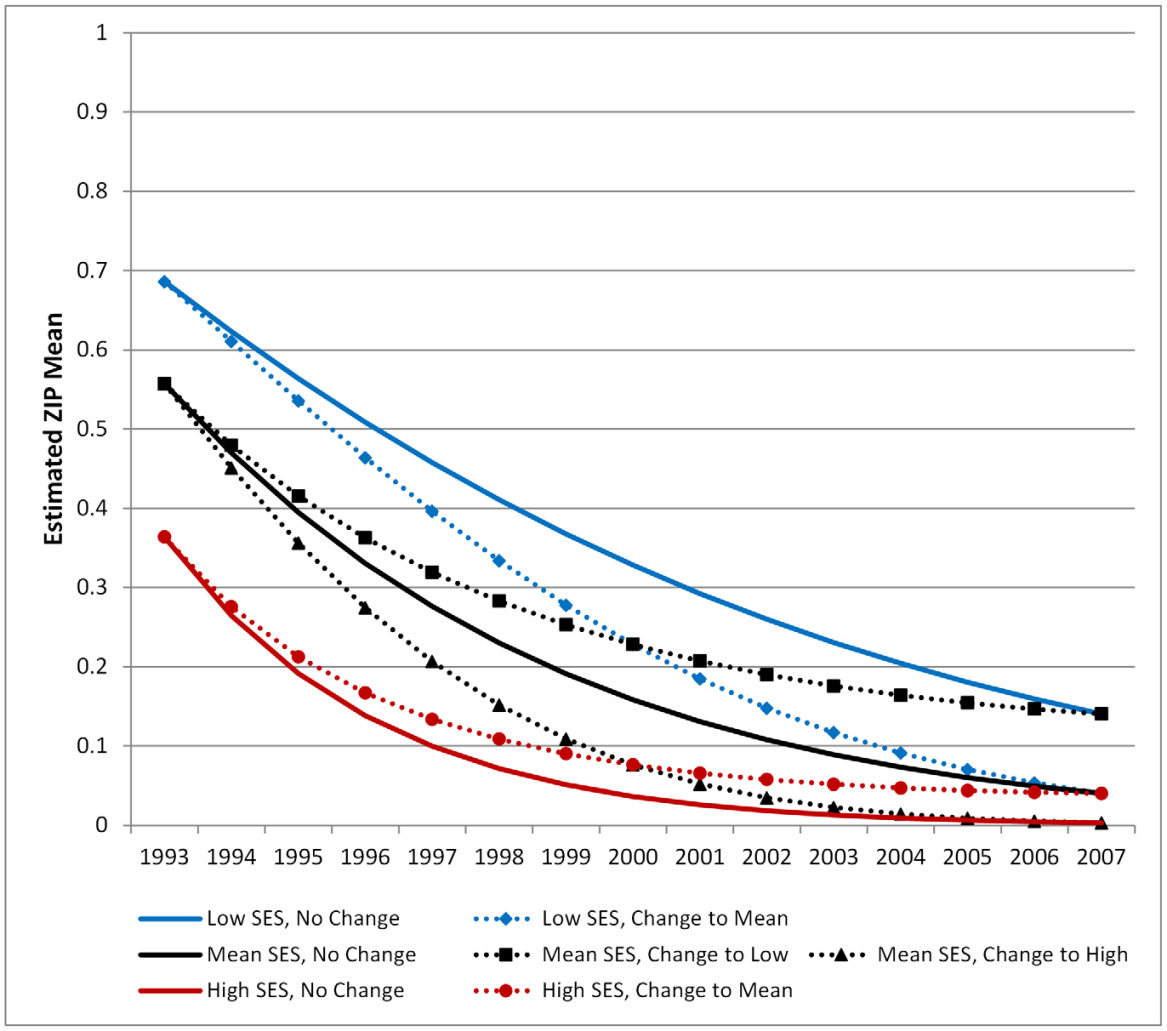
Mean ZIP cholera trajectories for different initial SES groups. Line graph indicating the mean trajectory of cholera cases from the full ZIP model (Model C) for different levels of SES. These trajectories were estimated holding population constant at 40 people per *bari* and distance from the icddr,b hospital at 5 km. The blue lines model the trajectory of cholera for *baris* with low initial SES (1 SD below the mean), the black lines model cholera for *baris* with mean initial SES, and the red lines model cholera for *baris* with high initial SES (1 SD above the mean). Solid lines indicate trajectories for *baris* where the level of SES stays constant over the study period. Dotted lines indicate trajectory for *baris* where the level of SES either increases or decreases over time. The figure legend indicates how SES changes over time.

The key covariate of interest in this analysis, SES, provides the most interesting findings from the study. The addition of SES has an effect on the initial number of cholera cases in a *bari* at the beginning of the study period (intercept) but not on the trajectory of change over time (slope). On average, *baris* with a high SES had lower initial rates of cholera (*γ_01_* = −0.147, p = 0.041) and a higher probability of reporting no cholera cases (*α_01_* = 0.156, p = 0.061), though these results were only marginally significant. The rate of decline in cholera cases (the slope) was not significantly affected by SES, though the coefficient for the Poisson model was in the expected direction (negative). For example, *baris* with a higher initial SES experienced rates of change that decelerate with time, suggesting that the overall rate of change was slower than baris with a lower initial SES. Figure 3 shows the estimated mean cholera trajectories for the Poisson portion of the model for baris with a population of 50 that are 5 km from the ICDDR,B hospital. Three different values of SES were selected for illustration – the mean, and one standard deviation above and below the mean. The figure clearly demonstrates the difference in the initial count of cholera cases – ranging from 1.85 cases for the lowest SES *bari* to 1.25 for the highest SES bari – and the overall decrease in cholera cases over time across all levels of SES. Figure 3 also demonstrates how the time variant SES measure modifies the slope of the trajectory over time. The decrease in cholera cases for a *bari* with a low initial SES score that stays low over the study period (solid blue line), is less than the decrease in cases for a *bari* with a low initial SES that improves over time (dotted blue line, diamonds). This relationship holds true across all levels of initial SES. A *bari* with an initial SES at the mean that experiences lower SES over time (black dotted line, squares) will initially see a decrease in the number of cholera cases, which increases toward the end of the study period. Figure 4 shows the estimated mean trajectories for the combined ZIP model (both the Poisson and ZI models together).

The random effect for initial status declines by 54.8% from Model A to Model C suggesting the additional variables explain a significant portion of the between-*bari* variance in cholera counts. Because it is still statistically significant, potentially explainable residual variation in initial status remains. The random effect for the slope declines by 30.4% from Model A to Model C, though it too is still statistically significant, suggesting the presence of potentially explainable residual variation in rates of change. Since the effect of SES on time is not statistically significant, but the variance component is, this indicates that SES does modify the slope for some baris, but not all.

## Discussion

The longitudinal nature of the cholera data that has been systematically collected in Matlab over the past 28 years coupled with the wealth of longitudinal demographic, economic and geographic data available for the population, allows for the innovative analysis of the effect of a variety socio-demographic factors on cholera dynamics over time. This study is unique in two respects: first it examines the effect of *bari*-level socioeconomic status on the longitudinal change in cholera and second it identifies the role of SES during the introduction of a new biotype of cholera into Bangladesh. Earlier studies only examined the change in cholera over time, without considering other socio-demographic factors [13], [29], or did not apply longitudinal methods to examine the impact of socio-demographic factors on change over time [6], [7], [30]. As such, this study extends our understanding of the effects of *bari*-level socioeconomic status on cholera rates over time.

Our analysis shows that socioeconomic status had a significant impact of the initial number of cholera cases in a *bari* at the beginning of the 0139 epidemic, net of all other covariates. However, since SES had no discernible effect on the rate of change over time, SES does not appear to play a strong role in the rate at which cholera cases decline to pre-epidemic levels. This is not surprising given that the decline in cholera is due to a variety of factors, including the population gradually building immunity to the disease. Therefore, it is likely that we do not have the necessary data to properly model the decline in cases over time.

SES is an indicator of several factors that directly impact cholera transmission, namely sanitation and education. Income (measured by assets here) allows households to purchase adequate housing, upgrade sanitation systems and improve drinking water. Higher SES households are more likely to have a latrine with a septic system (i.e., cement ring latrines with septic holding tank) or a deep tube well, both of which protect household members from fecal-oral contamination. When several households in a *bari* are able to afford such improvements, risk of cross-household contamination is even less. This is consistent with an earlier study by Emch [12] which found that cholera is more common in poorer households with less access to tube well water and sanitary latrines. High SES households are also likely to have a higher overall level of education, which can also impact cholera dynamics. The mechanism by which education affects cholera risk is not well understood, but may be related to improved personal hygiene or the increase in income often associated with better education. In this study we created an index of SES which included not only household wealth, but also education and sanitation. In our analyses, we found that household assets and sanitation (e.g., latrine and drinking water source) were so highly correlated (r = 0.712; p<0.0001) that including both as individual variables decreased the effect of both indicators. Households with better economic prospects invest in improved sanitation systems. However, models with sanitation variables only did not fit as well as models with the SES variable. We found a similar effect with education – wealthier households appear to invest in educating family members. Our findings served to reinforce the concept that SES is a multi-dimensional concept that should be measured using a variety of social and economic variables. Our findings were robust, and models using asset-only SES, with and without education and sanitation, showed the same association between SES and cholera occurrence as the final model presented in this paper.

Women's education in particular is often strongly correlated with child health [31]–[33], including diarrheal disease [34]–[36]. The pathways by which maternal education leads to better child health are still under investigation, but researchers have suggested that higher levels of education lead to improved care seeking behavior and use of medical care, proper hygiene and a better understanding of the causes of diarrheal illness among children [37]–[39]. There is, however, some evidence to suggest that this relationship is attenuated by other individual and household socioeconomic characteristics, such as income, sanitation, and marital status [40]–[42] or that maternal education is protective only in socioeconomically advantaged communities [43]. In this study we chose to examine the role of education in conjunction with other factors which contribute to household SES rather than focus solely on the role of women's education. We did this for several reasons. First, our analysis is not limited to cholera cases among children - we include adolescents and adults with cholera - and the link between health and women's education is strongest when predicting child morbidity. Since the average age of individuals with an O139 case was significantly higher than the average for El Tor cholera (possibly due to the lack of natural immunity in the population), there are many adult cholera cases during the time period included in this study. Second, our modeling strategy does not estimate individual risk of cholera; rather it estimates the combined risk of cholera for all individuals in a *bari*. If education is a predictor of cholera risk, then we felt we must consider the average level of education attained by members of the *bari* for which we are predicting cholera cases, not just the educational levels of women. Finally, single variable measures of education were highly collinear with SES Indices, creating problems with model estimation.

Since the SES index explained more of the variation in cholera than the sanitation variable alone, we suggest that there is some aspect of high socioeconomic status, above and beyond simply improving sanitation, which affects cholera risk. We are not able to say from our models the exact mechanisms by which high SES reduces cholera risk, but we hypothesize it may be due to a combination of education, hygiene knowledge and practices, better housing quality and access to clean drinking water. The finding that SES modifies the initial effect of the *V. cholera* 0139 epidemic is remarkable given that most of the population in rural Bangladesh is very poor. This study shows disparities exist in cholera transmission even among the very poor. Populations in *baris* characterized by low SES are more likely to experience higher cholera morbidity at the beginning of an epidemic than populations in high SES *baris*. The policy implication of this finding is that local level poverty alleviation programs which include improvements to sanitation and drinking water access - *as well as other strategies to improve overall SES* - will likely have an impact on cholera, especially during the introduction of new biotypes.

## Supporting Information

Checklist S1 **STROBE checklist.**
(DOC)

Table S1 **Additional model specifications with random slope and intercept terms for both the Poisson and ZI portions of the ZIP model.**
(XLS)

Table S2 **Additional model specifications showing robusteness check of SES indices, sanitation variable and age.**
(XLS)
