# Supplementary material for: The Role of Socioeconomic Status in Longitudinal Trends of Cholera in Matlab, Bangladesh, 1993–2007
Source: PLoS Negl Trop Dis. 2013 Jan 10;7(1):e1997. doi: 10.1371/journal.pntd.0001997 (PMC3542183; doi:10.1371/journal.pntd.0001997)
Supplement: Checklist S1 — STROBE checklist. (DOC) [file pntd.0001997.s001.doc]

STROBE Statement—checklist of items that should be included in reports of observational studies

|  | | Item No | Reported in Section, Paragraph | | |
| --- | --- | --- | --- | --- | --- |
| **Title and abstract** | | 1 | Abstract | | |
| Introduction | |  |  | | |
| Background/rationale | | 2 | Introduction, Paragraph 1/2 | | |
| Objectives | | 3 | Introduction, Paragraph 5 | | |
| Methods | |  |  |  | |
| Study design | | 4 | Methods | | |
| Setting | | 5 | Methods: Study Area/Data, Paragraph 1/2/3 | | |
| Participants | | 6 | Methods: Data, Paragraph 2/3/4  Also, readers can refer to reference [24] and [29] | | |
| Variables | | 7 | Methods: Data/SES Measurement/Statistical Analysis, Paragraph 2/4/5/6/10 | | |
| Data sources/ measurement | | 8 | Methods: Data/SES Measurement, Paragraph 2/3/4/5  Also, readers can refer to reference [23] and [24] | | |
| Bias | | 9 | N/A | | |
| Study size | | 10 | Methods: Data, Paragraph 4 | | |
| Quantitative variables | | 11 | Methods: Data, SES Measurement, Paragraph 3/5, Table 1 | | |
| Statistical methods | | 12 | Methods: Data/SES Measurement/Statistical Analysis, Paragraph 3-5/8-11 | | |
| Results | | | | |  |
| Participants | | 13 | Methods: Data, Paragraph 2/4 | | |
| Descriptive data | | 14 | Table 2 | | |
| Outcome data | | 15 | Methods: Data, Paragraph 4, Table 1 | | |
| Main results | | 16 | Table 3 | | |
| Other analyses | | 17 | Methods: SES Measurement, Supplementary Tables | | |
| Discussion | | | | | |
| Key results | 18 | | Discussion, Paragraph 2/3 | | |
| Limitations | 19 | | Discussion, Paragraph 2/3 | | |
| Interpretation | 20 | | Discussion | | |
| Generalisability | 21 | | Discussion, Paragraph 5 | | |
| Other information | | | | | |
| Funding | 22 | | Provided online | | |
